# Migratory bats are attracted by red light but not by warm‐white light: Implications for the protection of nocturnal migrants

**DOI:** 10.1002/ece3.4400

**Published:** 2018-08-25

**Authors:** Christian C. Voigt, Katharina Rehnig, Oliver Lindecke, Gunārs Pētersons

**Affiliations:** ^1^ Leibniz Institute for Zoo and Wildlife Research Berlin Germany; ^2^ Institute of Biology Freie Universität Berlin Berlin Germany; ^3^ Faculty of Life Science University of Vienna Vienna Austria; ^4^ Faculty of Veterinary Medicine Latvia University of Life Sciences and Technologies Jelgava Latvia

**Keywords:** animal orientation, aviation lighting, bat migration, conservation, light pollution, phototaxis, wind turbines

## Abstract

The replacement of conventional lighting with energy‐saving light emitting diodes (LED) is a worldwide trend, yet its consequences for animals and ecosystems are poorly understood. Strictly nocturnal animals such as bats are particularly sensitive to artificial light at night (ALAN). Past studies have shown that bats, in general, respond to ALAN according to the emitted light color and that migratory bats, in particular, exhibit phototaxis in response to green light. As red and white light is frequently used in outdoor lighting, we asked how migratory bats respond to these wavelength spectra. At a major migration corridor, we recorded the presence of migrating bats based on ultrasonic recorders during 10‐min light‐on/light‐off intervals to red or warm‐white LED, interspersed with dark controls. When the red LED was switched on, we observed an increase in flight activity for *Pipistrellus pygmaeus* and a trend for a higher activity for *Pipistrellus nathusii*. As the higher flight activity of bats was not associated with increased feeding, we rule out the possibility that bats foraged at the red LED light. Instead, bats may have flown toward the red LED light source. When exposed to warm‐white LED, general flight activity at the light source did not increase, yet we observed an increased foraging activity directly at the light source compared to the dark control. Our findings highlight a response of migratory bats toward LED light that was dependent on light color. The most parsimonious explanation for the response to red LED is phototaxis and for the response to warm‐white LED foraging. Our findings call for caution in the application of red aviation lighting, particularly at wind turbines, as this light color might attract bats, leading eventually to an increased collision risk of migratory bats at wind turbines.

## 1 INTRODUCTION

Globally, average light emissions in outdoor environments grow at a rate of 6% per year, which has unforeseen and poorly understood consequences for the biodiversity of ecosystems (Davies & Smyth, 2017; Hölker, Wolter, Perkin, & Tockner, 2010; Kyba et al., 2017; Rich & Longcore, 2013). Natural light is an important driver of physiological processes in animals and a cue for orientation in the environment. Light controls circadian rhythms, and thus behavior, or it may affect ecological networks by altering predator–prey interactions (e.g., Davies & Smyth, 2017; Manfrin et al., 2018; Rich & Longcore, 2013; Rowse, Lewanzik, Stone, Harris, & Jones, 2016). The consequences of artificial light at night (ALAN) on animals are known since the early days of city illumination at the turn of the 19th century (Rich & Longcore, 2013). For example, it is a well‐established fact that insects get lured by street light and then might die at the light source because of collision, exhaustion, or predation. One of the most prevalent vertebrate predators at street lights is bats. In Europe, fast‐flying and agile bats of the genera *Eptesicus*,* Nyctalus*, and *Pipistrellus* are considered to be relatively light‐tolerant (Rowse et al., 2016; Stone, Harris, & Jones, 2015). By contrast, slow‐flying species such as those of the genus *Myotis* exhibit more light averse behavior, possibly because slow flight makes them more vulnerable to predation by visually oriented sit‐and‐wait predators such as owls. Thus, the effect of ALAN on bats seems to vary according to the presence of profitable insect accumulations and species‐specific escape behaviors defined by the specific motion capacity. Past studies have also highlighted that light intensity as well as wave length spectra trigger different responses to light sources in bats (Lewanzik & Voigt, 2016; Spoelstra et al., 2015, 2017). Spoelstra et al. (2015) observed a higher activity of *P. pipistrellus* at green light sources. In a more detailed study, each of the recorded species showed a specific response to ALAN light according to the dominant wave length of the light source (red, green, and white). Slow‐flying bats of the genera *Plecotus* and *Myotis* avoided white and green light, but did not show a specific response to red light compared to darkness. By contrast, species of the genus *Pipistrellus* were more often recorded close to white and green light, yet these bats did not exhibit a specific response to red light. The authors suggested that attraction to white and green light was caused by bats hunting insects which were attracted to light of these wave length spectra (Spoelstra et al., 2017). Almost all previous studies focused on bats during the nonmigration period. In the first study on migratory bats, Voigt, Roeleke, Marggraf, Pētersons, and Voigt‐Heucke (2017) showed that bats are attracted to green light when migrating. Specifically, Voigt et al. (2017) showed that the activity of the two most migratory species *Pipistrellus nathusii* and *P. pygmaeus* increased by more than half when being exposed to green light sources compared to darkness when flying along a major migration corridor at the shoreline of the Baltic Sea in Latvia. This response behavior was independent of hunting activity and thus resembled phototaxis.

Here, we studied the effect of red and white LED on wild migratory bats, because these light sources are more commonly used for outdoor lighting than the previously studied green light. Indeed, ALAN based on these two wave length spectra is commonly found in almost all anthropogenic environments, particularly in urban areas. Some of the light sources have probably only a local effect on organisms, for example, when the light cone is directed toward a road, a bike trail, or a path. Yet, some ALAN can be seen from distant places. For instance, the skyglow of urbanized areas is visible over relatively long distances (Falchi et al., 2016). Other far‐reaching light sources are more pointed and less diffuse than the sky glow. For example, the white light beam of lighthouses or the red aviation lighting on top of tall buildings, towers, and wind turbines has far‐reaching light beams which often emit light in all directions. In fact, red signal lighting is globally obligate nowadays and applied for sea, land, and airspace since the 1950s and 1960s (e.g., Breckenridge, 1967). Usually, bats are exposed to all of these light sources when migrating in the open space, yet it is unknown if they exhibit a wavelength‐specific response to ALAN during their annual journeys, particularly to red light sources, which could put migratory bats at risk when getting attracted to dangerous tall structures like wind turbines (Voigt, Currie, Fritze, Roeleke, & Lindecke, 2018).

In Europe, long‐distance migration of Nathusius’ bats (*P. nathusii*), Soprano bats (*P. pygmaeus*), Common noctule bats (*Nyctalus noctula*) can be observed in August and early September along the Eastern shorelines of the Baltic Sea (Ijäs, Kahilainen, Vasko, & Lilley, 2017; Pētersons, 1990, 2004). Some of these species can travel up to 2,000 km from their summer roosts to their wintering grounds and are exposed to a wide range of illuminated environments when migrating over Central and Western Europe (Pētersons, 1990, 2004). Here, we used the same experimental setup at the migration corridor in Latvia as described by Voigt et al. (2017), but switched the light source from green wave length spectra to LED emitting light in the red and white spectrum. The wavelength composition in the red spectrum was similar to conventional red aviation light installed globally (Breckenridge, 1967). Specifically, we installed three poles with ultrasonic recorders along a 46 m transect line rectangular to the flight direction of migrating bats. One pole was erected at each end of this line and one pole in the center. The central pole carried a white board that was illuminated by LED light of one specific type (red or warm‐white) in 10‐min intervals. If migratory bats are attracted toward red or warm‐white LED, we expected to record a higher activity of bats directly at the light source. If this attraction is related to feeding activity, we expected to record more so‐called feeding buzzes when the light treatment was switched on compared to dark control periods at the central pole carrying the light source. Feeding buzzes are stereotypic repetitions of echolocation calls (EC) that indicate an insect hunt. If a possible attraction is not related to feeding activity, we would not expect an effect of light on the frequency of feeding buzzes. If migratory bats are repelled by the light sources, we would expect a lower activity of bats directly at the light source, that is, the central pole, and a higher activity at the lateral poles at about 23 m distance to the central pole.

## 2 MATERIALS AND METHODS

We conducted our experiment at the Pape Ornithological Research Station in Southwest Latvia (56°10′N 020°55′E) under the licences Nr.31/2016‐E from 6 July 2016 and Nr.33/2017‐E from 19 July 2017 issued by the Latvian Nature Conservation agency. The station is part of the Pape Nature Park and located between the Pape Lake and the Baltic Sea Coast and according to a recent survey not affected largely by diffuse skyglow (Falchi et al., 2016). Each year, thousands of bats pass the coastal corridor during summer migration in August and early September (Pētersons, 1990; Rydell et al., 2014; Steffens et al., 2004). Migratory bats commonly observed at this site are *P. nathusii*,* P. pygmaeus*,* N. noctula* and *Vespertilio murinus*. Nonmigratory bats such as *Plecotus auritus*,* Myotis brandtii*,* M. nattereri*,* Eptesicus nilssonii* also occur (Pētersons, 1990).

### 2.1 Experimental setup

We performed our study between 10 August and 6 September 2016 at an open area (meadow) next to the Heligoland trap of the station. Three 8 m high poles were set up at a distance of 23 m from one another along a line that was rectangular to the shoreline, and thus rectangular to the migration direction of bats. The westward pole (seaside) was at 100 m distance from the Baltic Sea. Each pole was equipped with an Electret Ultrasound Microphone (Avisoft Bioacoustics/Knowles FG, Berlin, Germany) at a height of 5.3 m above the ground. The microphones were pointing northward, opposite to the flight direction of the migratory bats to ensure that all EC of passing bats were recorded. On the central pole, we mounted a white plastic board (0.4 × 4 m) at 4 m above the ground. We equipped this board with two parallel LED lines each consisting of eight strips of warm‐white LED and eight strips with red LED lights. Each strip carried 28 LED (revoART ^®^ e.K., Borsdorf, Germany). The lateral poles were lacking any light source. We measured the spectral compositions of the LED with a spectrometer (type CAS140CT, OSA Opto light GmbH, Berlin). The red LED had a dominant wavelength at 623 nm and a peak wavelength at 631 nm. The luminous intensity was measured as 0.4695 cd and the radiant intensity as 0.002426 W/sr. For the warm‐white LED, we recorded a dominant wavelength at 581 nm and a peak wavelength at 576 nm. The luminous intensity equalled 0.4894 cd and the radiant intensity 0.001314 W/sr (see ESM for spectra of both light sources). Luminous intensity was measured with a luxmeter (VOLTCRAFT LX‐1108, Conrad GmbH, Germany) at a distance of 5 m from the central pole and at a height of 1.5 m. We calculated a value of 1.8 ± 0.19 lx (mean ± standard deviation) for the red and a value of 3.3 ± 0.17 lx for the warm‐white LED light.

The illumination was programmed to start at dusk (9:30 pm) and to stop at dawn (5:30 am), with an alternating on/off‐rhythm every 10 min. The light‐off intervals were used as a dark control. Whenever the light was switched on, the acoustic recorder received a low‐frequency analogous signal so that the light treatment was visible on the ultrasonic recordings. Each night was divided into two halves of equal lengths during which we tested one of the two LED color. During subsequent nights, we switched the sequence of LED colors (first red, then white, or first white, then red). Bat EC were recorded with the Avisoft – RECORDER USGH software (Avisoft Bioacoustics, Berlin, Germany), which triggered the 3s recordings following an ultrasonic signal above 19 kHz.

### 2.2 Acoustic analysis

For quantifying the response behavior of migratory bats, we focused on the period between 19 August and 6 September 2016 to ensure that the majority recordings included migratory and not resident bats. This period is hereafter referred to as peak migration. Three nights were excluded from further analysis because the recordings were not activated due to technical problems or advert ambient conditions (heavy rainfall and strong winds). Furthermore, we excluded the 29 August 2016 from further statistical analysis because of low flight activity (22 EC in total for this night). We used Avisoft‐SAS Lab Pro (Aviosoft Bioacoustics, Berlin, Germany) bioacoustics software for analyzing EC. In a first step, we eliminated all sound files with erroneously‐recorded noises, such as insect calls or background noises in the ultrasonic range. In a second step, bat species were identified based on their characteristic EC using the automatic bat species identification tool in Avisoft‐SAS Lab Pro. For the automated assignment of bat species, we selected EC of species frequently observed at our study site. We used the open access library of ecoObs GmbH (http://www.ecoobs.de; Anonymous 2010) as a reference. We focused on 27 characteristic features of EC (such as length of call, dominant frequency, range of frequency, call interval, among others) for *P. nathusii*, 24 for *P. pygmaeus*, four for *P. auritus*, seven for *M. brandtii*, six for *M. nattereri*, 10 for *N. noctula* and eight for *N. leisleri* as well as eight for *E. nilssonii* and 10 for *V. murinus*. To avoid incorrect species identification (Russo & Voigt 2016), we confirmed and, if necessary, corrected all identified calls manually by visual inspection of the recordings. We calculated the cumulative number of EC for each microphone (pole), each light spectrum and each light‐on/light‐off‐interval. As the call characteristics of species of the genera *Nyctalus*,* Eptesicus* and *Vespertilio*, and those of the genus *Myotis* are very similar, we could not distinguish species of these genera. Hence, we grouped all identified species of these genera in the group of *Nyctaloid* and species of the genus *Myotis* in the group *Myotis*. Due to a very limited number of recorded EC of bats of the *Myotis* group and of *Barbastella barbastellus* and *P. auritus*, we excluded those from further statistical analysis.

### 2.3 Statistical analysis

All statistical analyses were conducted using R 3.3.3 (R.app GUI 1.69 (7328 Mavericks build), S. Urbanek& H.‐J. Bibiko, © Team, R.C., 2016), using the following packages: glmmADMB (Bolker, Skaug, Magnusson, & Nielsen, 2012), bbmle (Bolker & Bolker, 2017) and glmulti (Calcagno, Calcagno, Java, & Suggests, 2017). To correct for the effects of varying weather conditions on the flight activity of migrating bats, a generalized linear model (GLM) was fitted. The total number of ECs per night was set as response variable. We log‐transformed data to meet the assumptions of normality. The predictor variables were set to the experimental days (1–19) as well as the average of temperature (°C), daily rainfall (mm), wind speed (m/s), wind direction (0–360°, 16 segments) and lunar phase (converted in the lighting percentage [%]) of each 8 hr experimental night. Weather data was recorded every hour by the meterological station DAVIS Vantage PRO2 Wireless at Pape. An automated model selection was performed with the R package glmulti (Calcagno et al., 2017) and the GLM was fitted with the predictors (day, temperature, daily rainfall and wind speed) as well as a Gaussian distribution and identity link function. To test the explanatory predictors of interest a likelihood ratio test was performed. The significant level (*p*‐value) was calculated using the chi‐square test (*χ* ^2^) distribution. We used an alpha value of 5% for this and all following statistical tests.

We conducted generalized linear mixed models (GLMMs) to measure the influence of the two different light treatments on bat activity during peak migration. The GLMM was fitted with the package glmmADMB (Generalized Linear Mixed Models Using AD Model Builder, version 0.8.3.3) to handle excess zeros (zero‐inflation) and overdispersion (Bolker et al., 2012; Zuur, Ieno, Walker, Saveliev, & Smith, 2009). For each of the 10 min intervals, we defined the cumulative EC count numbers of each species/species group as the response variable in the GLMM. The experimental days (1–19) were set as random effects and treatment {light‐on (white/red [1/2], light‐off [0])} as well as night half (first/second [1/2]) were set as predictor variables. The Akaike Information Criterion (AIC) was used to fit the best model in glmmADMB (Burnham & Anderson, 2003). The model was fitted with a Poisson, binomial or negative binomial distribution and zero‐inflation true/false. The R package bbmle was used to construct the AIC for model comparisons (Bolker & Bolker, 2017). Then, the effect of light treatment on flight activity was calculated with a Wald chi‐square test.

To investigate whether the effect of light is correlated with a higher insect density or a positive phototaxis, we calculated the ratio between the number of FBs and the total number of EC for each of the three species/species group at the central pole to account for a higher likelihood of detecting a feeding buzz when more bats are present. Then, we performed a Wilcoxon Test for each LED type to test for significant differences in feeding activity between light‐on and light‐off periods. This analysis was performed only for recording from Nathusius’ pipistrelle, as we did not record enough feeding buzzes for any of the other groups.

## 3 RESULTS

### 3.1 General migration activity of bats during late summer

**Figure 1:**
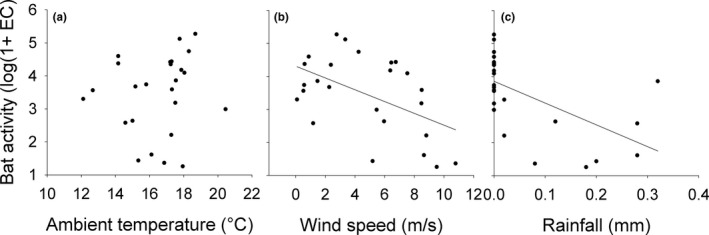
Correlation between mean number of echolocation call (EC) per night (log(1 + EC)‐transformed) and mean ambient temperatures (°C; a), mean wind speed (m/s; b) and daily rainfall (mm; c)

In total, we counted 570,340 EC at all three poles over the course of the study. On the day of highest activity (20 August 2016), we observed a total number of 186,398 EC, followed by the second highest number of recordings (131,453 EC) shortly afterward on the 22 August 2016. The median of daily EC recordings for the whole study period equalled 4,747 EC (range: 17–186,398). Three climate factors were suggested by the model as predictors for bat activity: temperature (°C), daily rainfall (mm) and wind speed (m/s). The model did not reveal an influence of wind direction and lunar cycle on migration activity, and therefore, these factors were excluded from further analysis. A GLM detected a correlation of the total number of EC with the day (*χ* ^2^ = 28.37, *p* < 0.001) and a negative effect of daily rainfall (*χ* ^2^ = 22.14, *p* < 0.001) and wind speed (*χ* ^2^ = 9.7, *p* < 0.01), but not of ambient temperature (*χ* ^2^ = 22, *p* = 0.572, Figure 1) on migration activity.

At the seaside pole, we observed 55% of EC (313,199), that is, the majority of flight activity (median: 3,012; range: 2–94,459), followed by the landside pole with a total of 139,489 EC (24%; median: 802; range: 0–63,148), and lastly the central pole with a total of 117,652 EC (21%; median: 1,034; range: 0–34,530). We identified *P. nathusii* as the most common species (490,533 EC; 86% of all EC), followed by bats of the *Nyctaloid* group (64,739 EC; 11.4%) and *P*. *pygmaeus* (14,005 EC; 2.4%). A small proportion of EC originated from bats of the *Myotis* group (897 EC; 0.3%), from *P. auritus* (135 EC; 0.02%) and from *B. barbastellus* (31 EC; 0.01%).

An interaction between night half and flight activity did not turn significant for any species/species group during the red light treatment (*P. nathusii*:* χ* ^2^ = 0.35, *df* = 1, *p* = 0.56; *n* = 16; *P. pygmaeus: χ* ^2^ = 0.03, *df* = 1, *p* = 0.86; *n* = 14; *Nyctaloid*:* χ* ^2^ = 0.01, *df* = 1, *p* = 0.93; *n* = 15) as well as during the white light treatment (*P. nathusii*:* χ* ^2^ = 0.66, *df* = 1, *p* = 0.42; *n* = 16; *P. pygmaeus*:* χ* ^2^ = 1.82, *df* = 1, *p* = 0.18; *n* = 9; *Nyctaloid*:* χ* ^2^ = 0.55, *df* = 1, *p* = 0.46; *n* = 14). Thus, we neglected the timing of the treatment (first or second half of night) in the further analyses.

### 3.2 Red LED treatment

**Figure 2:**
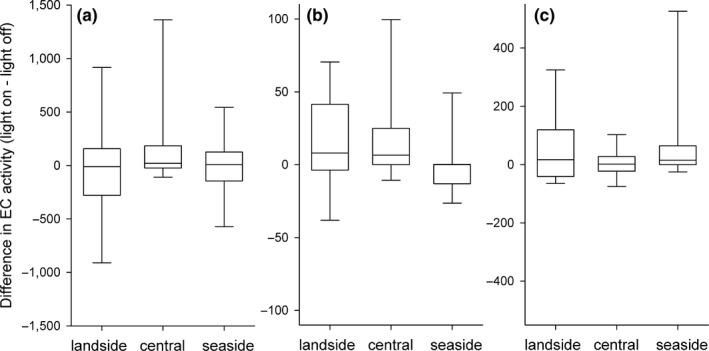
Differences in echolocation call (EC) activity during peak migration for *Pipistrellus nathusii* (a), *P. pygmaeus* (b) and the *Nyctaloid* group (c) for the seaside pole, the central pole with red LED illumination and the landside pole. Positive values indicate a higher activity during the illuminated periods

We recorded at the central pole a median of 457 EC per night for *P. nathusii* (total: 34,927; range: 0–13,842), 17 EC per night for *P. pygmaeus* (total: 922; range: 0–405) and 43 EC per night for bats of the *Nyctaloid* group (total: 5,782; range: 0–3,565) when red LED light was switched on. Compared to the dark control, EC activity increased by 73% in *P. pygmaeus* when red LED was switched on (Table 1). *Pipistrellus nathusii* showed a trend for a higher activity when red LED was switched on (Table 1). We were not able to record a difference in EC activity for bats of the *Nyctaloid* group between lit and unlit periods (Table 1; Figure 2).

**Table 1:** Generalized linear mixed model evaluating the effect of red and warm‐white light emitting diodes (LED) light on the activity of migratory bats (*Pipistrellus nathusii*,* P. pygmaeus* and species of the *Nyctaloid* group)

Species	Pole	Red LED		Warm‐white LED	
		*p*‐Value	*z*‐Value	*p*‐Value	*z*‐Value
*P. nathusii*	Seaside	0.62	0.5	0.64	2.94
	Central	***0.08***	1.77	0.56	−0.58
	Landside	0.16	1.39	**<0.01**	2.73
*P. pygmaeus*	seaside	0.10	1.63	0.83	−0.22
	Central	**0.03**	2.24	0.70	0.39
	Landside	0.98	−0.02	0.19	1.31
*Nyctaloid*	Seaside	0.76	0.31	0.68	1.31
	Central	0.35	1.22	0.25	1.15
	Landside	**<0.01**	2.79	0.71	0.37

*Note*

Significant results are highlighted in bold and trends in bold and italic. Pole 1 = seaside, pole 2 = central pole with LED source, pole 3 = landside pole).

At the seaside lateral pole, we counted a median of 2,406 EC per night for *P. nathusii* (total: 76,550; range: 10–27,264), 46 EC per night for *P. pygmaeus* (total: 1,974; range: 0–864) and 80 EC per night for the *Nyctaloid* group (total: 11,800; range: 0–6,787) during the light‐on treatment. Here, we did not observe an effect of the treatment on flight activity for any species/species group (Table 1). At the landside lateral pole, we counted a median of 426 EC per night for P. nathusii (total: 38,943; range: 0–22,251), 0 EC per night for P. pygmaeus (total: 745; range: 0–434) and 37 EC per night for bats of the *Nyctaloid* group (total: 7,159; range: 0–4,713) during the light‐on treatment. We did not observe a difference in activity between treatments for *P. nathusii* or for *P. pygmaeus* (Table 1), yet we recorded more EC for bats of the *Nyctaloid* group at the landside pole when the LED light was switched on compared to darkness (Table 1).

### 3.3 Warm‐white LED treatment

**Figure 3:**
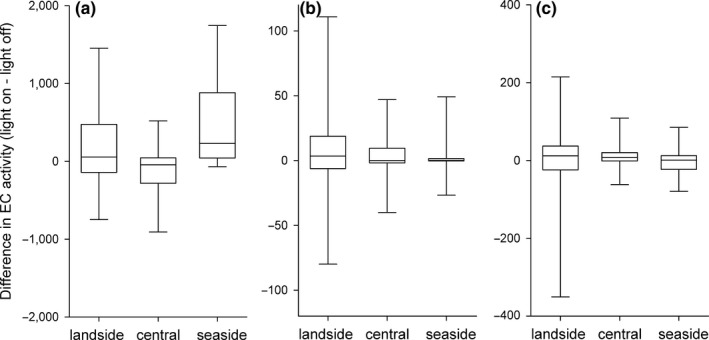
Differences in echolocation call (EC) activity during peak migration for *Pipistrellus nathusii* (a), *P. pygmaeus* (b) and the *Nyctaloid* group (c) for the seaside pole, the central pole with warm‐white LED illumination and the landside pole. Positive values indicate a higher activity during the illuminated periods

When warm‐white LED was switched on, we recorded at the central pole a median of 348 EC per night for *P. nathusii* (total: 17,375; range: 0–5,829), 0 EC per night for *P. pygmaeus* (total: 505; range: 0–125) and 25 EC per night for bats of the *Nyctaloid* (total: 2,161; range: 0–557). The analysis indicated no response of any species/species group to the LED light treatment at the central pole (Figure 3; Table 1).

At the seaside pole, we counted a median of 1,200 EC per night for *P. nathusii* (total: 60,748; range: 0–28,290), 24 EC per night for *P. pygmaeus* (total: 1,658; range 0–491) and 47 EC per night for bats of the *Nyctaloid* group (total: 4,365; range 0–1,666). We did not observe an effect of the light treatment on the number of EC in any species or group at the seaside pole (Table 1). At the landside pole, we observed a median of 498 EC per night for *P. nathusii* (total: 26,357; range: 0–5,639), 0 EC per night for *P. pygmaeus* (total: 753; range 0–347) and 23 EC per night for bats of the *Nyctaloid* group (total: 1,961; range: 0–465). The EC activity of *P. nathusii* was higher at the landside pole during the light‐on treatment, but not for the two other groups (Table 1; Figure 3).

### 3.4 Feeding activity at LED lights

In total, we recorded 139 feeding buzzes in 16 of the 17 recorded migration nights, most of which were produced by individuals of *P. nathusii* (*n* = 134 FB), followed by *P. pygmaeus* (*n* = 3) and the *Nyctaloid* group (*n* = 2). Except for hunting activity of *P. nathusii*, feeding buzzes of all other bat species were too rare in order to conduct statistical testing. For the red LED treatment, we did not observe a significant difference in the number of feeding buzzes in relation to the overall EC activity for *P. nathusii* between the light‐on treatment compared to the dark control (*Z* = 29, *n* = 8 nights, *p* = 0.12). For Nathusius’ bats, we confirmed a higher relative feeding activity when exposing bats to warm‐white LED compared to darkness (*Z* = 20, *n* = 6 nights, *p* < 0.05).

## 4 DISCUSSION

We tested if European bats, namely *P. nathusii*,* P. pygmaeus* and species of the *Nyctaloid* group, were attracted to or repelled by red or warm‐white LED during late summer migration. Two factors could cause a possible attraction toward the displayed LED light sources: First, bats could be attracted toward it because of disorientation after exposure to specific light wavelengths. Second, bats could be attracted to LED light because of foraging insects lured by the light source. During the red LED treatment, we observed an increased EC activity in *P. pygmaeus* and a trend for higher activity in *P. nathusii*. Both increased EC activities were not associated with an increased hunting activity. For warm‐white LED light, we did not observe an increased EC activity for any of the species. Although hunting activity was similarly low at warm‐white LED compared to red LED light, we recorded an increase in the number of feeding buzzes in *P. nathusii* when the warm‐white LED was switched on compared to the dark control.

### 4.1 Migration phenology and species composition

In general, migration activity varied largely between days during the experimental period. Flight activity of bats correlated negatively with daily rainfall and wind speed, but not with ambient temperature. Major migration movements began directly after the day of full moon on the 19 August 2016, with highest migration activities recorded during the following days. Based on ultrasonic recordings, we identified five species of bats and two species groups (*Nyctaloid* and *Myotis*) which consisted of several species with similar EC features. The species composition was dominated by P. nathusii (86% of all recorded EC), which confirms previous findings that this species is the most frequent migratory bat in northeastern Europe (Ijäs et al., 2017; Pētersons, 2004; Rydell et al., 2010; Voigt et al., 2016, 2017). About 11% of recorded EC originated from bats of the species group Nyctaloid, which includes most likely *N. noctula*,* V. murinus* and *E. nilssonii*; four bat species commonly captured in the nearby Heligoland trap of the station. About 2% of all recorded EC were emitted by *P. pygmaeus*; a species that is not well recognized as a migratory species in the literature, yet which shows a profound migration phenology at our study site.

### 4.2 Response of migratory bats to red LED light

For the red LED treatment, we recorded significantly more EC of *P. pygmaeus* at the central pole during the light‐on compared to the light‐off treatment. *Pipistrellus nathusii* showed a trend for an increased EC activity during the light‐on treatment. The positive effect of red LED light on EC activity for *P. pygmaeus* was as strong as the response of this species and *P. nathusii* to green light in a previous experiment (about 50% increase, Voigt et al., 2017). The significant findings for *P. pygmaeus* are noteworthy, as at our study site, *P. pygmaeus* is less abundant than *P. nathusii* (2% vs. 84% of all recorded EC for *P. pygmaeus* and *P. nathusii*, respectively). Therefore, the significant positive effect was detected with a far lower number of individuals in *P. pygmaeus* compared to the trend observed for *P. nathusii* and the red light treatment.

The overall low hunting activity of migratory bats was similar to observations from the previous study (Voigt et al., 2017). Nathusius’ bats did not hunt insects at the central pole when the red LED light was switched on. This is not surprising as most insect taxa are rather attracted to light of short wavelengths, such as ultraviolet light, and not necessarily to light of the red wavelength spectrum (Rich & Longcore, 2013 Van Grunsven et al., 2014; Van Langevelde, Ettema, Donners, Wallis DeVries, & Groenendijk, 2011). The fact that migratory bats captured at the study site appear to have already consumed insects (Krüger, Clare, Symondson, Keišs, & Pētersons, 2014; Voigt, Sorgel, Suba, Keiss, & Pētersons, 2012) could best be explained by migratory bats feeding prior to launching into the skies for nightly migration flight; a behavior that could best be explained by the phrase “forage first, migrate afterwards.” In bats of the *Nyctaloid* group we did not observe a response toward the red LED light at the central pole, but a significant increase in activity at the landside pole. As the *Nyctaloid* group includes not only potentially migratory species such as *N. noctula*,* N. leisleri* and *V. murinus*, but also nonmigratory and locally abundant species, namely *E. nilssonii*, it is difficult to interpret the results, as we do not know which of the lumped species caused the positive effect.

Previous studies on light spectra specific response of bats highlighted that bats do not respond to red light in any specific way, irrespective of their taxonomic affiliation (Spoelstra et al., 2015, 2017). In the study by Spoelstra and colleagues, species of the genus *Pipistrellus* were not separated and thus it is difficult to compare our findings with those of Spoelstra and colleagues. However, it is surprising that the attraction effect of red LED light on *P. pygmaeus* and to a lesser extent on *P. nathusii* at the Latvian migration corridor stands in contrast to the findings of Spoelstra et al. (2017). We argue that this difference is not caused by methodological differences or different experimental designs, but rather by condition‐dependent effects of ALAN on bats before and during the migration period. It is noteworthy in this context that a context‐specific response to light cues has also been discussed for compass orientation of bats, that is, nonmigratory bats used solar cues for orientation, whereas migratory bats did not (Greif, Borissov, Yovel, & Holland, 2014; Lindecke, Voigt, Pētersons, & Holland, 2015). We speculate that migratory bats may be more susceptible to light sources of specific wave length spectra because vision may play a more dominant role than echolocation during migration. Nonmigratory bats might use orientation cues that are more involved during general hunting behavior, for example, echoes reflected from local landmarks, instead of cues from natural or artificial light sources.

### 4.3 Response to warm‐white LED light

We did not observe an effect of warm‐white LED light on the acoustic activity of the two species of *Pipistrellus* and of bats of the *Nyctaloid* group at the central pole. An increased acoustic activity of *P. nathusii* at the landside pole during illuminated periods could possibly be explained by some avoidance behavior when the white LED was switched on. Yet, the higher feeding activity of *P. nathusii* at the central pole during lit periods argues against an avoidance of *P. nathusii* in response to warm‐white light. Spoelstra et al. (2017) recorded more bats of the genus *Pipistrellus* under white light conditions than under darkness, indicating that species of this genus might hunt opportunistically for insects around white light sources. In our experiment, the relatively high number of feeding buzzes emitted by Nathusius’ bats in the presence of warm‐white LED indicated hunting behavior, even though warm‐white LED light is considered less attractive for insects than cold‐white LED (Eisenbeis & Eick, 2011; Wakefield, Broyles, Stone, Jones, & Harris, 2016).

### 4.4 General discussion and conservation implications

The results of this experiment support an earlier study conducted with green light at the same study site that ALAN, in general, affects the acoustic activity and most likely flight behavior of migratory bats, namely *P. nathusii* and *P. pygmaeus*. The lack of insect hunting at the red and green light sources indicates that the attraction of migratory bats to light sources of these wavelength spectra was not caused by foraging. The most likely explanation for the observed higher activity of migratory of bats at red and green light sources is a light‐dependent “fixed direction” response (Wiltschko, Stapput, Thalus, & Wiltschko, 2010; Wiltschko & Wiltschko, 2009). Alternatively, migrating bats use a sensory modality for long‐distance navigation that is vulnerable to light of specific wavelengths, yet the underlying mechanisms, particularly of a light‐dependent magnetic sense, needs to obtain more empirical support for bats in particular and mammals in general. It is the current understanding that mammals have a cryptochrome (cry1) that has not yet proven to be an effective part of the magnetic sense, nor has it been found in bats in particular (Nießner et al., 2016). Irrespective of the mechanisms underlying the fixed direction response, it is important to consider the consequence of our findings for conservation management. Red light sources are frequently used for safety reasons, for example, to prevent airplanes or helicopters from crashing into tall structures or for guiding boats and ships along distinct routes close to the shoreline. Particularly, aviation lighting on top of wind turbines might cause a fatal attraction over kilometers when bats may fly toward the light source and then collide with the operating rotor blades, yet we lack comprehensive studies in this direction for European bat species (Ballasus, Kill, & Hüppop, 2009; Bennett & Hale, 2014). Migratory bats are by far the species with the highest collision risk at wind turbines (Rydell et al., 2010, Voigt et al. 2012, 2015) and our study suggests that this pattern might be influenced by the use of aviation lighting on top of wind turbines. We argue that bat‐friendly lighting, such as in the infrared range, which is also promoted by pilots for safety reasons, or context‐dependent operation of aviation lighting at wind turbines might present a way to mitigate the negative effects of ALAN on migratory bats at wind turbines. Yet, further studies testing light sources in the infrared wavelength spectrum, particularly on top of tall structures, need to be conducted before formulating general management recommendations.

## CONFLICT OF INTEREST

None declared.

## AUTHOR CONTRIBUTIONS

CCV developed the concept and experimental design; CCV ensured funding; KR and OL conducted the experiment; GP contributed to field work; KR analyzed the data; All authors discussed the data; CCV wrote a draft of the manuscript; All other authors commented and edited the draft.
