# Supplementary material for: Migratory bats are attracted by red light but not by warm‐white light: Implications for the protection of nocturnal migrants
Source: Ecol Evol. 2018 Aug 25;8(18):9353–61. doi: 10.1002/ece3.4400 (PMC6194273; doi:10.1002/ece3.4400)
Supplement: Supplementary file 1 [file ECE3-8-9353-s001.docx]

**Figure S1.** Radiant intensity (W/sr nm) for red LED light (A) and warm-white LED light (B) used in the experiments.
